# Successful mandible rehabilitation of lower incisors with one-piece implants

**DOI:** 10.1186/1752-1947-8-406

**Published:** 2014-12-05

**Authors:** Dorina Lauritano, Roberto Grassi, Dario di Stasio, Alberta Lucchese, Massimo Petruzzi

**Affiliations:** 7 Department of Neuroscience and Biomedical Technologies, University of Milan Bicocca Monza, Via Cadore n° 48, 20052 Monza, MB Italy; 8 Dental School, Section of Oral Pathology and Medicine, University of Bari, Bari, Italy; 9 Multidisciplinary Department of Medical and Dental Specialties, Second University of Naples, Naples, Italy

## Abstract

**Introduction:**

The popularity of one-piece implants has increased considerably among patients and dentists. The advantages of one-piece immediate loading are to reduce the number of interventions. These parameters can be better controlled with a one-piece implant.

**Methods:**

We considered 21 patients with one-piece implants inserted in mandible for this retrospective study. Inclusion criteria were: good oral hygiene, absence of lesions of the oral mucosa, no smoking or smoking less than 20 cigarettes a day, drinking less than two glasses of wine a day, good general health and no pregnancy.

**Results:**

We enrolled 21 (12 women and 9 men) patients in this retrospective study. The mean follow-up was 1 year. A total of 84 one-piece implants were inserted in mandible to replace 42 lower first and 42 second incisors. The diameter of the implants was 3.0mm in all fixtures. The length of the implants was equal to or longer than 12mm in 44 and 40 fixtures respectively. Of these, 48 were inserted in women and 36 in men (age range 33 to 67; mean age 58.3 years).

**Conclusions:**

There is no difference between the survival rates of one-piece immediate loading implants and two-piece implants and delayed loading. In conclusion, a one-piece immediate loading implant is a reliable device for mandible rehabilitation.

## Introduction

The popularity of immediate loading implants has increased considerably among patients and dentists [1]. The advantages of immediate loading are to reduce the number of interventions and time of prosthetic [2]. Furthermore, the success of immediate loading is related to the primary implant stability and loading control [3]. In fact, primary stability has always been considered fundamental to osseointegration. To facilitate the immediate loading protocol, the stability of the implant at the time of placement is essential [4], and implant surface modifications have a significant role in measuring the success of osseointegration. The primary implant stability at placement is the mechanical phenomenon related to the quality and quantity of the bone at the recipient site, the type and design of the implant used. These parameters can be better controlled with a one-pieiece implant [4]. In fact, although two-piece implants have shown great success for a long time, the two stages of surgical procedures, the infiltration of bacteria in the microgap between abutment and implant, and the screw fracture after loading, are considered complications that could be overcome by the use of one-piece implants [5]. In addition, the one-piece implant allows a minimally invasive flapless surgery which is very well accepted by patients [6]. The immediate prosthetic of a one-piece system allows for better tissue healing [7], better adhesion of the gingival mucosa to form a collar which is healthy and adherent to the implant, and avoiding a second surgical procedure [8]. The prosthetic procedure of a one-piece implant enables the physiology of the natural tooth. The one-piece implant enables a borderline preparation following the contour of the gingival margin leading to a better preservation of mucous seal [9]. One-piece immediate loading implants have a survival rate similar to delayed loading implants [10]. Since immediate loading of one-piece implants has become a widely used procedure for rehabilitation of partially edentulous patients, we decided to perform a retrospective study on one-piece implants inserted in partially edentulous mandibles.

## Results

We enrolled 21 (12 women and 9 men) patients in this retrospective study. The mean follow-up was 1 year. A total of 84 one-piece implants (BioHorizons, Italy) were inserted in the mandible to replace 42 lower first and 42 second incisors. The diameter of the implants was 3.0mm in all fixtures. The length of the implants was equal to or longer than 12mm in 44 and 40 fixtures respectively. Of these, 48 were inserted in women and 36 in men (age range 33 to 67 years; mean age 58.3 years; Table 1).

**Table 1:** **Number of implants inserted in 12 women and 9 men and length of implants**

Implants	12mm	>12mm	Total	Lost
Women	23	25	48	4
Men	21	15	36	6
Total	44	40	84	10

Ten implants were lost after 1 year follow-up. The implant survival rate (ISR) was 80.5%.

Peri-implant bone reabsorption was investigated in the remaining 74 implants. Only two implants have more than 1.5mm of crestal bone reabsorption after 1 year.

## Discussion

In the literature there are no studies describing one-piece immediate loading implants to restore incisors in the lower jaw with a median follow-up of 1 year. Early publications on one-piece implants for restoration of lower first and second incisors sites were presented as case reports [11, 12]. One-piece immediate loading implants have been becoming a widespread procedure for oral rehabilitation of edentulous jaws. Many factors can influence the implants’ survival: surgical technique, bone quality, the type of implants, and system factors related to the occlusion. The surgical technique should provide for the primary stability; bone quality and quantity are also key factors for implant success [13]. The type of implant includes design, surface characteristics, and diameter and length of implant. Occlusion is related to prosthetic design [14]. Patient’s compliance is better with a one-piece implant than with a two-stage procedure. In fact patients experience pain and inflammation more frequently with a traditional delayed prosthetic. The osseointegration of a two-piece implant needs 3 to 6 months before loading, so the prosthetic phase can begin. In addition, patients who have a flapless procedure need minimal post-surgery medications, and flapless surgery allows blood supply to new bone reducing reabsorption. A single stage with one-piece implants provides a better osseointegration avoiding micromovements of the fixture, a good soft tissue healing and less discomfort for the patient [15].

## Conclusion

The limitation of the study is that there is no comparison available between the survival rate of the one piece and the two piece. However, there is no difference between the survival rate of one-piece immediate loading implants and two-piece implants and delayed loading. The mandible has a good bone quality and good bone quality is a predictor of implants’ survival rate [15]. A one-piece implant design avoids manipulating soft gingival peri-implant tissues, allowing adhesion of connective tissue to bone and implant-abutment neck. In conclusion, a one-piece immediate loading implant is a reliable device for mandible rehabilitation. The advantages of one-piece implants are manifold: functional and esthetic rehabilitation, reduced operating time, less armamentarium, less damage to surrounding tissue, and a better use of the space where the bone thickness is reduced. The management of partial edentulous space requires a multidisciplinary approach. Implant success depends on the amount and quality of bone tissue and the primary stability that allows a correct osseointegration. Patient compliance to oral rehabilitation is definitely promoted by one-piece implants with careful planning and a minimally invasive technique.

In conclusion, the solution of complex implantology problems, as in post-extractive implantology with immediate load, requires an adequate competence and an accurate diagnostic process of feasibility, to select the ideal implant for this type of implantology application.

## Methods

### Study design

#### Sample

A total of 21 patients with one-piece implants inserted in the mandible were considered for this retrospective study. The study population was selected from the patients of three dentists working in private practice and treated with implant therapy from 2012 to 2014. The inclusion criteria were: good oral hygiene, absence of lesions of the oral mucosa, no smoking or smoking less than 20 cigarettes a day, drinking less than two glasses of wine a day, good general health (no prior chemotherapy or radiotherapy; no immunosuppression or corticosteroid therapy; no liver, blood or kidney disease), and no pregnancy. All patients signed an informed consent for participation in the study. This study was approved by local committee of Republic of San Marino (trail registration number AB571479456).

### Outcomes

The first outcome was ISR 1 year after insertion and prosthetic. The second outcome was the peri-implant bone reabsorption (PBR). It was evaluated in relation to reabsorption of bone of more than 1.5mm in the first year after implant insertion.

### Data collection

All patients were subjected to a radiographic X-ray (Sirona Orthophos XG 5, Siemens, Italy) with software for image processing (Sidexis XG, Siemens, Italy). A computed tomography scan (Gendex, KaVo Italia) before implants insertion was performed when necessary. Peri-implant crestal bone levels were evaluated by the calibrated examination of radiographic X-ray after surgery and at the end of follow-up period (1 year). The measurements were carried out medially and distally to each implant calculating the distance between the implant neck and the most coronal point of contact between implant and bone. The measure was put in relation to the level of bone measured immediately after the insertion of the implant, which was the point of reference for subsequent measurements. The radiographs with measures were saved in a compressed TIFF format. Each file was processed with a Microsoft Windows XP professional operating system using Photoshop 8.0 (Adobe, USA) and shown on a 432mm (17-inch) Asus computer display. The Sidexis software allows radiographic measurements corresponding to the real ones. The difference between the implant abutment junction and the bone crestal level was defined as PBR.

### Surgical protocol

**Figure 1:**
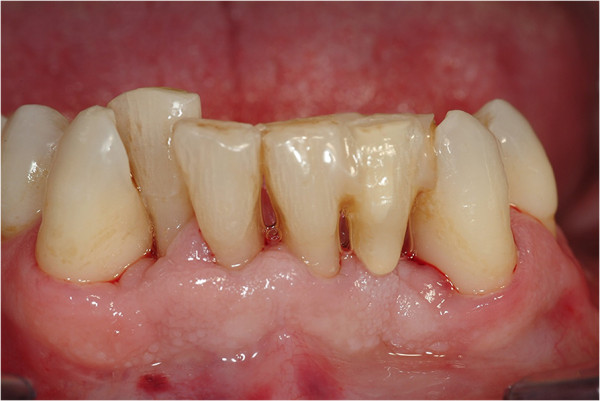
**Splinted lower incisors in periodontal patient.**

**Figure 2:**
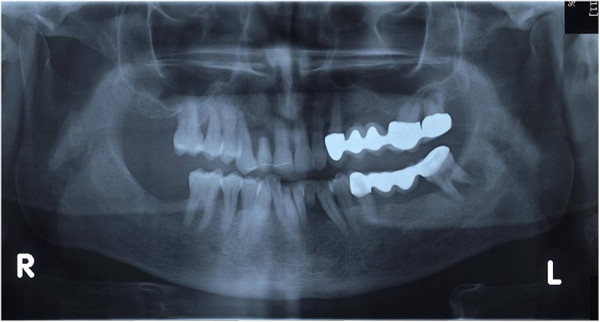
**Panoramic X-ray showing vertical bone loss.**

**Figure 3:**
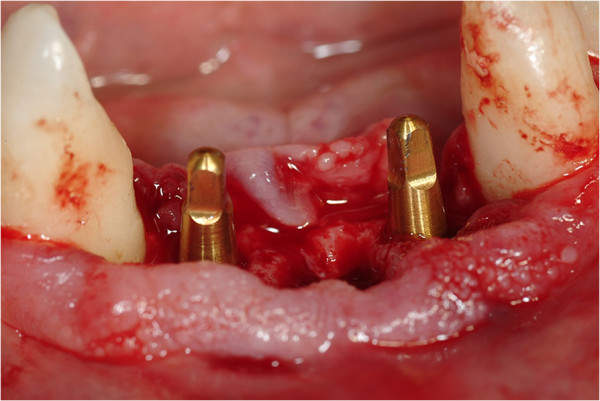
**One-piece implants placed in fresh extraction sites.**

**Figure 4:**
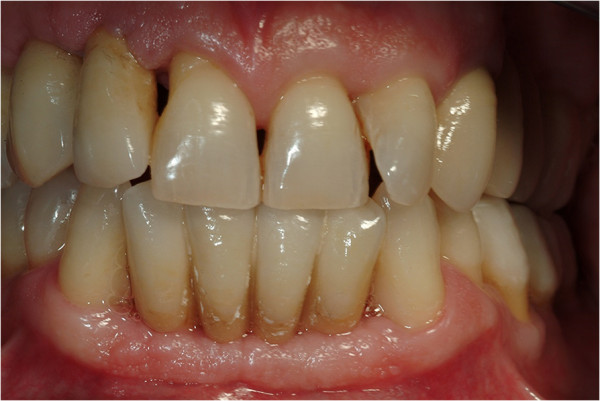
**Prosthetic rehabilitation after 4 weeks.**

**Figure 5:**
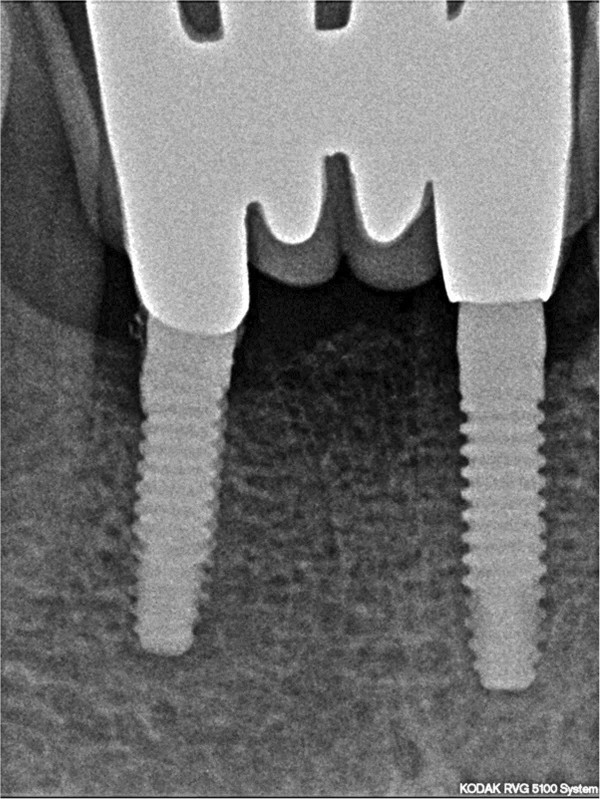
**Panoramic X-ray after 1 year follow-up.**

All patients followed the same surgical protocol (Figures 1 and 2).The anesthesia of the jaw was obtained by the injection of articaine and post-surgical analgesic treatment was performed with 100mg of ketoprofen three times a day if necessary. One-piece implants (BioHorizons, Italy) were inserted in fresh extraction sites (Figure 3). All implant necks were positioned at the crestal level. A provisional prosthesis was provided within 48 hours and a definitive within 4 weeks (Figures 4 and 5). All patients agreed to follow a strict oral hygiene protocol and recall.

T-test analysis was used to calculate ISR and PBR.

## Abbreviations

ISR: Implant survival rate
PBR: Peri-implant bone reabsorption.
